# Supplementary material for: The causal relationship between the human gut microbiota and pyogenic arthritis: a Mendelian randomization study
Source: Front Cell Infect Microbiol. 2024 Nov 26;14:1452480. doi: 10.3389/fcimb.2024.1452480 (PMC11629706; doi:10.3389/fcimb.2024.1452480)
Supplement: Supplementary file 1 [file DataSheet1.zip › mendelian test/ebi-a-GCST90027571.csv_leave_one_out.pdf]

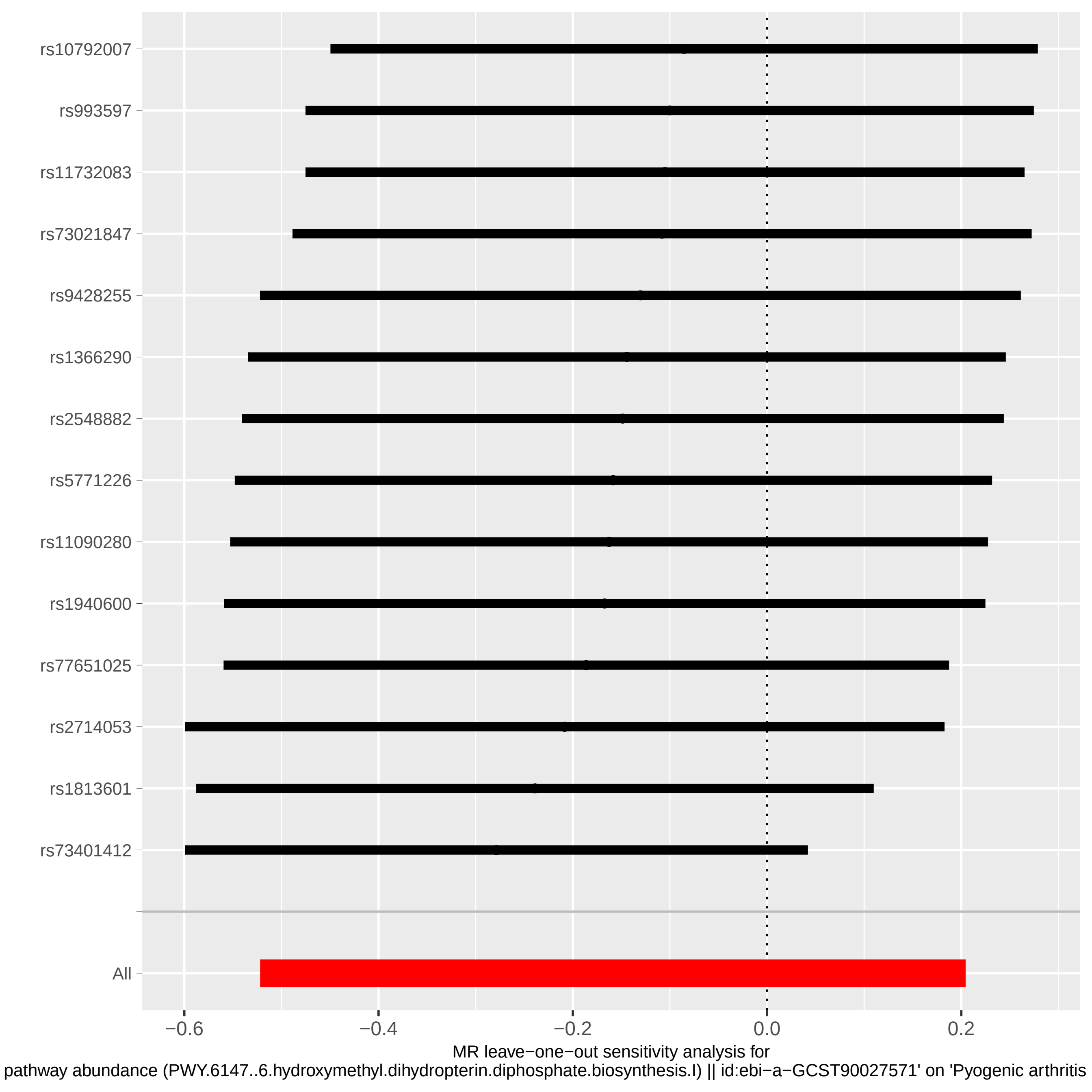
rs10792007
rs993597
rs11732083
rs73021847
rs9428255
rs1366290
rs2548882
rs5771226
rs11090280
rs1940600
rs77651025
rs2714053
rs1813601
rs73401412
All
−0.6
−0.4
−0.2
0.0
0.2
MR leave−one−out sensitivity analysis for
pathway abundance (PWY.6147..6.hydroxymethyl.dihydropterin.diphosphate.biosynthesis.I) || id:ebi−a−GCST90027571' on 'Pyogenic arthritis
